# Development and characterization of 33 novel polymorphic microsatellite markers for the brown tree snake *Boiga irregularis*

**DOI:** 10.1186/s13104-015-1620-z

**Published:** 2015-11-07

**Authors:** Shem D. Unger, Erin F. Abernethy, Stacey L. Lance, Rochelle R. Beasley, Bruce A. Kimball, Thomas W. McAuliffe, Kenneth L. Jones, Olin E. Rhodes

**Affiliations:** Savannah River Ecology Laboratory, University of Georgia, Aiken, SC 29802 USA; United States Department of Agriculture, Animal and Plant Health Inspection Service, Wildlife Services, National Wildlife Resources Center, Monell Chemical Senses Center, 3500 Market Street, Philadelphia, PA 19104 USA; United States Department of Agriculture, Animal and Plant Health Inspection Service, Wildlife Services, National Wildlife Research Center, P.O. Box 10880, 210 Amau’ulu Road, Hilo, HI 96720 USA; Department of Biochemistry and Molecular Genetics, University of Colorado School of Medicine, Aurora, CO 80045 USA

**Keywords:** *Boigairregularis*, Brown tree snake, Invasive species, Illumina, Microsatellites

## Abstract

**Background:**

*Boiga irregularis* is a widespread invasive species on Guam and has led to extirpation of most of the island’s native avifauna. There are presently no microsatellite markers for this invasive species, hence we developed highly polymorphic microsatellite markers to allow for robust population genetic studies on Guam.

**Findings:**

We isolated and characterized 33 microsatellite loci for the brown tree snake, *B. irregularis*. The loci were screened across 32 individuals from Guam. The number of alleles per locus ranged from three to ten, with an average of 4.62. The expected (H_e_) and observed heterozygosity (H_o_) ranged from 0.294 to 0.856 and from 0.031 to 0.813, with an average of 0.648 and 0.524, respectively. Significant deviations from Hardy–Weinberg equilibrium were detected at seven loci after Bonferoni correction. Probability of identity values ranged from 0.043 to 0.539.

**Conclusions:**

These genetic markers are useful for understanding a suite of post-invasion population genetic parameters, sources of invasions, and effectiveness of management strategies for this invasive species.

**Electronic supplementary material:**

The online version of this article (doi:10.1186/s13104-015-1620-z) contains supplementary material, which is available to authorized users.

## Findings

The brown tree snake *Boiga irregularis* was inadvertently introduced to the island of Guam following World War II [1] and has devastated native avifauna. There is presently only one study examining the genetic composition of source populations using DNA sequence data from mitochondrial and nuclear sequence loci [2]. Therefore, highly polymorphic multilocus markers are needed to investigate the genetic ramifications of invasive species demography and population genetics of *B. irregularis*.

To investigate the potential use of population genetics tools to inform invasive species management by investigating source populations or determining the current population structure of *B. irregularis* on Guam, we developed microsatellite markers for *B. irregularis*. Genomic DNA used to isolate the microsatellite loci was extracted from one individual, utilizing a QIAamp DNA microkit (QIAGEN). An Illumina paired-end shotgun library was prepared by following the standard protocol of the Illumina nextera DNA sample preparation kit and using a dual index identifier adaptor. Illumina sequencing was conducted on the HiSeq with 100 bp (base pair) paired-end reads. The DNA libraries were prepared to range from 300–600 bp and then paired end reads were performed on the resulting fragments. Thus we sequenced 100 bp from end to end, with primers designed on the reads (one from each) as a majority of the time the two sequences did not overlap, leaving an unknown length of unsequenced bases between the reads. Using the program PAL_FINDER_v0.02.03 [3], 190,804 reads containing tetra, penta, and hexanucleotide repeats were identified and batched to a local installation of the program Primer3 (version 2.0.0) for primer design.

Forty-eight primer pairs were tested for amplification and polymorphism following the methods detailed in O’Bryhim et al. [4]. Amplification reactions in 12.5 µl contained 20 ng of genomic DNA, 10 mM Tris pH 8.4, 50 mM KCl, 25.0 µg/ml BSA, 0.4 µM unlabeled primer, 0.04 µM CAG-tag labeled primer, 0.36 µM universal dye-labeled primer, 3.0 mM MgCl_2_, 0.8 mM dNTPs, using an applied biosystems GeneAmp 9700. The forward primer from each locus was 5′ modified with an engineered “CAG-tag” sequence to enable use of a third, fluorescently labeled primer in PCR following Schuelke 2000 [5]. Touchdown thermal cycling programs encompassing a 10 °C span of annealing temperatures ranging between 65 and 55 °C were used for all loci. Touchdown parameters consisted of an initial denaturation step of 5 min at 95  C followed by 20 cycles of 95 °C for 30 s, highest annealing temperature (decreased 0.5 °C per cycle) for 30 s, and 72 °C for 30 s; and 20 cycles of 95 °C for 30 s, lowest annealing temperature for 30 s, and 72 °C for 30 s, and a final extension at 72 °C for 5 min. PCR products were run on an ABI-3130xl sequencer and sized with Naurox size standard prepared as described in DeWoody et al. [6], except that the unlabeled primer for the Naurox standard was GTTT pig-tailed. Results were analyzed using GeneMapper version 3.7 (Applied Biosystems). Thirty-three of the tested 48 primer pairs amplified high quality PCR product that exhibited polymorphism and consistent allele base pair amplification.

We assessed the variability of the 33 polymorphic loci for 32 individuals from Guam. Conditions and characteristics of the loci are provided in Addditional file 1: Table S1. Cervus version 3.0.6 was used to calculate the number of alleles per locus (*A*) and observed and expected heterozygosity (H_o_ and H_e_) [7]. All 33 loci were determined to be polymorphic, with a range of 3–10 alleles per locus with an average of 4.62 (Additional file 1: Table S1). Probability of identity (PI) was estimated using GenAlEx v6.5 [8]. Deviations from Hardy–Weinberg equilibrium (HWE) and linkage disequilibrium were performed using GENEPOP v4.0 [9]. After Bonferroni correction for multiple comparisons, eight loci showed significant deviations from HWE (α = 0.00139), and linkage disequilibrium was detected for 1 of the 630 paired loci (α = 0.0000794). Probability of identity values ranged from 0.043 to 0.539. These new microsatellite loci are ideally suited to address important questions including testing the efficacy of management and control techniques for *B*. *irregularis* on Guam by providing markers ideal for assessment of population structure before and after eradication efforts.

## Availability of supporting data

The microsatellite sequences are available through the National Centre for Biotechnology Information (see http://www.ncbi.nlm.nih.gov/); GenBank accession no SRR2033931 (submitted 5/20/2015, made public 08/3/2015).

## Additional file

10.1186/s13104-015-1620-z Details for 33 polymorphic microsatellite loci developed for the brown tree snake *Boiga irregularis* using 32 individuals.
