# Supplementary material for: Development and characterization of 33 novel polymorphic microsatellite markers for the brown tree snake Boiga irregularis
Source: BMC Res Notes. 2015 Nov 7;8:658. doi: 10.1186/s13104-015-1620-z (PMC4637142; doi:10.1186/s13104-015-1620-z)
Supplement: Supplementary file 1 — 10.1186/s13104-015-1620-z Details for 33 polymorphic microsatellite loci developed for the brown tree snake Boiga irregularis using 32 individuals. [file 13104_2015_1620_MOESM1_ESM.docx]

**Table S1** **Details for 33 polymorphic microsatellite loci developed for the brown tree snake *Boiga irregularis*** **using 32 individuals**

| Locus | Primer Sequence (5' to 3') Repeat motif | | Size (bp) | *A* | | H_o_ | | H_e_ | PI |  |
| --- | --- | --- | --- | --- | --- | --- | --- | --- | --- | --- |
| BOIR1 | F^a^: GTCCACCTATCCACCCACC | (ATCT)_16_ | 244-288 | 7 | | 0.594 | | 0.738 | 0.121 |  |
|  | R: AAAGATTGTCATTGGGAAAGC |  | |  | |  |  |  |  |  |
| BOIR4 | F^a^: AAATTGCCATCTTACTGCCC | (ATCT)_16_ | 292-316 | 6 | | 0.688 | | 0.716 | 0.129 |  |
|  | R: AAGCAGGAACCCACTACTCC |  | |  | |  |  |  |  |  |
| BOIR6 | F^a^: ATGTTTGCAAGCTTTGATGG | (AATAG)_14_ | 244-264 | 3 | | 0.531 | | 0.513 | 0.335 |  |
|  | R: AAGCTGTTGGGTTATTCCTCC |  | |  | |  |  |  |  |  |
| BOIR9 | F^a^: ACCACAAGGTGCTCCAATGC | (AAAG)_16_ | 286-352 | 9 | | 0.813 | | 0.789 | 0.080 |  |
|  | R: AGACCCTCAAGCTCTCGCC |  |  |  | |  | |  |  |  |
| BOIR10 | F^a^: GGAGCCGGAGGTGAGAGC | (ATCT)_19_ | 222-256 | 5 | | 0.563 | | 0.599 | 0.221 |  |
|  | R: AGGCAGCCTTCCTTCCAGG |  |  |  | |  | |  |  |  |
| BOIR11 | F^a^: CCTCTACGTCTACAGGATTCATTCG | (ATCT)_15_ | 282-302 | 6 | | 0.750 | | 0.737 | 0.123 |  |
|  | R: ATCTCCCACTTCCTGCCTGC |  |  |  | |  | |  |  |  |
| BOIR13 | F^a^: TTTGGTCTTTGCATAATGGG | (AGTCT)_15_ | 311-346 | 5 | | 0.563 | | 0.700 | 0.142 |  |
|  | R: CAATTCTCCAACACTGGACG |  | |  | |  |  |  |  |  |
| BOIR14^b^ | F^a^: AAGAGGCACAAAGGGACAGC | (AAAG)_17_ | 374-406 | 10 | | 0.469 | | 0.856 | 0.043 |  |
|  | R: CACATGATAGCAAGGAACTCCC |  | |  | |  |  |  |  |  |
| BOIR15 | F^a^: CTCTGGCTGATTTGCAGGG | (TTCC)_15_ | 332-348 | 4 | | 0.719 | | 0.681 | 0.178 |  |
|  | R: CACCTCCTTTCCTCCTTTCC |  |  |  | |  | |  |  |  |
| BOIR18 | F^a^: TTAGCAAAGAGAAGGAAGAGGC | (ATTTAC)_11_ | 261-327 | 5 | | 0.625 | | 0.757 | 0.110 |  |
|  | R: CATAGTATAGAGGGTGTATAGGCGG |  | |  | |  |  |  |  |  |
| BOIR19 | F^a^: GTCTGAAATGGATCGGTGGG | (AAGGAG)_11_ | 293-304 | 3 | | 0.563 | | 0.597 | 0.236 |  |
|  | R: CATTTCCCTTGTAAGAGTGGTGG |  | |  | |  |  |  |  |  |
| BOIR20 | F^a^: ACCGCTTCCTAGAGTTGGG | (AATAG)_16_ | 270-350 | 5 | | 0.750 | | 0.720 | 0.136 |  |
|  | R: CCATTTCGTCACCCTTCC |  |  |  | |  | |  |  |  |
| BOIR22^b^ | F^a^: CTGGAGAAACCACAGGCAGG | (TTCC)_15_ | 194-226 | 5 | | 0.281 | | 0.703 | 0.151 |  |
|  | R: CCCTCACAGGCTATTAAGCCC |  | |  | |  |  |  |  |  |
| BOIR23 | F^a^: GCAAATTCTACCCAATTCTCCC | (AAAG)_17_ | 270-442 | 4 | | 0.625 | | 0.711 | 0.139 |  |
|  | R: CCTCCTCTTTCTTTCCCTCC |  |  |  | |  | |  |  |  |
| BOIR24^b^ | F^a^: CTGCTTTCCTTGCACACAGC | (AAAG)_15_ | 228-242 | 4 | | 0.094 | | 0.510 | 0.290 |  |
|  | R: CGGAGGACTACAATGGCAGG |  | |  | |  |  |  |  |  |
| BOIR25 | F^a^: GGAAGTTCCTTCTATCACCAATGC | (TTCC)_16_ | 218-230 | 3 | | 0.531 | | 0.589 | 0.246 |  |
|  | R: CTGCTTAGAATTGAGGGCGG |  | |  | |  |  |  |  |  |
| BOIR26^b^ | F^a^: TTAAGCAGAGTTTGGCCTGG | (TTCC)_17_ | 272-284 | 3 | | 0.031 | | 0.294 | 0.539 |  |
|  | R: GAAAGCTGCTCAGTCATTGGC |  | |  | |  |  |  |  |  |
| BOIR27 | F^a^: CCTTCATCTGGTCAATCAGCC | (AAAG)_16_ | 174-198 | 4 | | 0.719 | | 0.616 | 0.227 |  |
|  | R: GAAGGGCTGCATACAAATCG |  | |  | |  |  |  |  |  |
| BOIR28 | F^a^: CAAATATATGAAACATTATCATGCAGAGG | (TTCC)_15_ | 262-282 | 4 | | 0.406 | | 0.534 | 0.279 |  |
|  | R: GACTATTAGGGATGTTTGCTGCC |  | |  | |  |  |  |  |  |
| BOIR29 | F^a^: AGCAGGCTCTGAAATTGTGC | (ATCT)_16_ | 270-282 | 3 | | 0.594 | | 0.514 | 0.344 |  |
|  | R: GATAGAGATAGAGGTGGGTGGG |  | |  | |  |  |  |  |  |
| BOIR30^b^ | F^a^: TGCATTGTTGGGCCATAGG | (TTCC)_17_ | 250-258 | 5 | | 0.031 | | 0.572 | 0.251 |  |
|  | R: GATGAATGGTAGGAGTCAATCAAGC |  | |  | |  |  |  |  |  |
| BOIR31 | F^a^: GGGATGGAGGAAAGGAGTCG | (AAAG)_17_ | 192-232 | 5 | | 0.375 | | 0.594 | 0.260 |  |
|  | R: GCAAACCAATCGGTGATGG |  |  |  | |  | |  |  |  |
| BOIR36^b^ | F^a^: GAAGGAGGATTGGTTGGAAGG | (TTCC)_18_ | 178-210 | 4 | | 0.656 | | 0.721 | 0.133 |  |
|  | R: GCTCGAGGTCACTCGTGAGG |  | |  | |  |  |  |  |  |
| BOIR37 | F^a^: AGGGTTCAGGTCATAGTCTTCC | (AATAC)_15_ | 305-340 | 4 | | 0.594 | | 0.719 | 0.137 |  |
|  | R: GGATGAAACTATTATCTTGACTGGG |  | |  | |  |  |  |  |  |
| BOIR38 | F^a^: GATTCATATACTGGACTCCAAATTGC | (ATATAC)_11_ | 313-349 | 4 | | 0.469 | | 0.607 | 0.228 |  |
|  | R: GGGCTTGGGATAGACATCTCC |  | |  | |  |  |  |  |  |
| BOIR39^b^ | F^a^: AGTCCGACCTTGGAGAAGGG | (AAAGAT)_11_ | 179-233 | 4 | | 0.406 | | 0.690 | 0.165 |  |
|  | R: GGTGGTGGTGTCTTTATTGGC |  | |  | |  |  |  |  |  |
| BOIR40 | F^a^: ATTCCATCCCACCAACTGC | (ACTCT)_16_ | 258-282 | 3 | | 0.469 | | 0.550 | 0.287 |  |
|  | R: GGTTCTCAATGCAACTCAAGG |  | |  | |  |  |  |  |  |
| BOIR41 | F^a^: GGGTTTAATCCAGAGATAAGTGGG | (AAAG)_16_ | 338-434 | 5 | | 0.781 | | 0.732 | 0.124 |  |
|  | R: TCCTTGGTAATCTGCTGTTTGG |  | |  | |  |  |  |  |  |
| BOIR43 | F^a^: CTTTGGGATCCATGGCTGG | (ATCT)_16_ | 401-417 | 5 | | 0.813 | | 0.717 | 0.139 |  |
|  | R: TCTGGTGCAATTCACTTATCGC |  | |  | |  |  |  |  |  |
| BOIR44 | F^a^: CGAACCACTACACCCACTCG | (AAAG)_16_ | 222-246 | 3 | | 0.656 | | 0.598 | 0.241 |  |
|  | R: TGAACATCATTATTATTCACTGACCC |  | |  | |  |  |  |  |  |
| BOIR45 | F^a^: AGTCTAGAACTAGGACACTGCTGC | (AAAG)_16_ | 367-387 | 6 | | 0.656 | | 0.664 | 0.167 |  |
|  | R: TGATGTAAGCAAAGGCTCACC |  | |  | |  |  |  |  |  |
| BOIR46 | F^a^: GGGCTCAATTCTGGTCATAAGC | (AAAG)_19_ | 211-243 | 5 | | 0.625 | | 0.684 | 0.168 |  |
|  | R: TGCCGAAAGAAAGAGGAAGG |  | |  | |  |  |  |  |  |
| BOIR48 | F^a^: GAAGTGAGATTATTGCAGCCAGG | (AAAG)_19_ | 293-305 | 3 | | 0.375 | | 0.667 | 0.193 |  |
|  | R: TTCTGAGAATGTTCTTCTTACTGCG |  | |  |  | |  |  |  |  |

The size indicates the range of observed alleles in base pairs and includes the length of the CAG tag; *A* is the number of alleles observed, H_o_ and H_e_ are observed and expected heterozygosity, respectively, and PI is the probability of identity.

^a^ indicates CAG tag (5’CAGTCGGGCGTCATCA-3’) label

^b^ indicates significant deviations from Hardy-Weinberg expectations after Bonferroni corrections
